# Towards Production of Cost-Effective Modification of SmCo_5_-Type Alloys Suitable for Permanent Magnets

**DOI:** 10.3390/ma17040808

**Published:** 2024-02-07

**Authors:** Margarit Gjoka, Charalampos Sarafidis, Stefanos Giaremis

**Affiliations:** 1 Institute of Nanoscience and Nanotechnology, National Centre for Science and Research “Demokritos”, 15341 Athens, Greece; 2 Department of and Physics, School of Sciences, Aristotle University of Thessaloniki, 54124 Thessaloniki, Greece

**Keywords:** SmCo_5_ magnet, intermetallic compounds, structure and magnetic properties

## Abstract

SmCo_5_ constitutes one of the strongest classes of permanent magnets, which exhibit magnetocrystalline anisotropy with uniaxial character and enormous energy and possess high Curie temperature. However, the performance of SmCo_5_ permanent magnets is hindered by a limited energy product and relatively high supply risk. Sm is a moderately expensive element within the lanthanide group, while Co is a more expensive material than Fe, making SmCo_5_-based permanent magnets among the most expensive materials in the group. Subsequently, the need for new materials with less content in critical and thus expensive resources is obvious. A promising path of producing new compounds that meet these requirements is the chemical modification of established materials used in PM towards the reduction of expensive resources, for example, reducing Co content with transition metals (like Fe, Ni) or using as substitutes raw rare earth materials with greater abundance than global demand, like Ce and La. Important instruments to achieve these goals are theoretical calculations, such as ab initio methods and especially DFT-based calculations, in predicting possible stable RE-TM intermetallic compounds and their magnetic properties. This review aims to present the progress of recent years in the production of improved SmCo_5_-type magnets.

## 1. Introduction

Moving away from fossil fuels is of great environmental, political, and economic importance. It is also a fact that energy transition is a materials transition, because all modern environmentally friendly technologies also need new, advanced materials. Also, the transportation infrastructures and whole energy system must become more efficient and sustainable. The most intense driving factors for permanent magnets are the transportation sector, energy production and other emerging markets like robotics [1]. Rare earths play a vital role in this transition scheme, and among all applications it is worth underlining the materials for permanent magnets, the most important element in the rare-earth market, at least for some of these elements. The permanent magnet market is divided into two major categories: high-performing and expensive rare-earth magnets and conventional, older materials based on other Fe compounds or transition metals like alnico or ferrites. A famous gap exists between these two categories; this gap is the focus of many research efforts because the potential discovery of materials that can be used in this area may release resources for high-performance applications while also improving the design of other applications that are now based on low-performing magnets [2]. For all these reasons, the expected demand for permanent magnets is difficult to meet from the current supply chain [3,4].

Perhaps the most important improvement in high- and medium-performing permanent magnets is the combination of materials supply risk and temperature endurance. High-temperature applications of the Nd-Fe-B system are limited due to the very low Curie temperature of the Nd_2_Fe_14_B main phase, just 588 K [5]. Minor improvements to achieve functionality in the vicinity of 450 °C require processing with heavy rare earths, mostly Dy and Tb, raw materials that are expensive and immensely scarce. On the other hand, permanent magnets based on SmCo_5_ have improved temperature resistance, with the main phase having a Curie temperature of 1020 K. Since the mid-sixties, Sm-Co permanent magnets have been the primary high-performance ones [6,7,8,9], and they still dominate high-temperature applications even today [10,11].

Permanent magnets are widely used for various applications like electric motors, sensors, magnetic separators, and magnetic resonance imaging (MRI) machines. The growing adoption of electric vehicles (EVs) is a major factor driving the demand for permanent magnets, mostly in EV motors. Additionally, the increasing focus on renewable energy, especially in offshore wind turbines, is providing further growth opportunities for the market [12].

Sm-Co based magnets are composed of Co, which is characterized as a critical material, and Sm [13,14]. Sm is a relatively expensive element within the lanthanide group. On the other hand, Co is much more expensive than Fe, making these alloys the most expensive class of materials used in permanent magnets. As a result, these magnets are economically viable, mainly in high-temperature applications where they cannot be replaced by other materials [15,16,17,18]. The cobalt price is expected to exceed 30,000 USD per metric ton in 2024 [19]. Iron is very abundant, and its cost is in general not a factor for consideration; however, the other transition metals like nickel and copper are not as cheap as iron. Copper is about 8500 USD per metric ton due to its importance as a low electrical resistance material in cables and wires. Nickel, a material important in applications like rechargeable batteries, is more expensive, but also its price presents extreme volatility, occasionally overpassing even cobalt [20]. Thankfully, the required amounts for substituting Co are not large, and this could provide a path towards sustainable improvements in SmCo_5_-type materials.

It is important to assess the current trends and prospects for the permanent magnet market and the relevant raw materials that are necessary. Permanent magnets account for 30% of global rare-earth demand, but for specific rare-earth minerals like Nd, Pr, Dy, and Sm, permanent market applications are the main market: these raw materials account for 25% of total rare-earth production, but 80% of global market value [21]. Environmental and geopolitical issues drive advanced societies towards decarbonizing their economies, especially the transportation sector, with electric mobility products like vehicles and bikes seeing exponentially increased sales [22]. Global electric vehicles stock accounts for more than 30 million units and presents a rapid increase expected to reach 100–140 million units in 2030; 95% of battery electric vehicles use motors based on rare-earth permanent magnets [23]. Although the large volume of permanent magnets used in vehicles belong to the Nd-Fe-B family, there are certain applications that require the temperature endurance of SmCo_5_ materials and the substitution strategies are in general the same in these systems, in general as described by C.C. Pavel et al. [24]. The improvement of rare-earth transition metal intermetallics in general can be utilized in a number of paths: element substitution, processing improvement, and engineering design. Engineering may play a vital role in materials’ selection, for example, the replacement of SmCo_5_-based materials in high-temperature applications with Nd-Fe-B-based magnets is not possible without proper cooling and vice versa. Also, apart from mobility, electricity generation depends on materials containing rare earths. Offshore wind generators are usually based on permanent magnets for construction simplicity since they do not require high rotation speed and they can be built without gearboxes (direct drive). It is expected that the specific advantages of direct-drive wind generators based on permanent magnets, i.e., light construction, better efficiency, especially at lower rotation speed, and lower maintenance cost, will increase their market share close to 50% by 2030 [25].

Geopolitical factors also play a vital role in product design and production, and this is perfectly illustrated in rare-earth raw materials. Studies [26,27] utilized the wavelet analysis method to study the correlation between rare-earth raw material prices and global geopolitical and economic factors. In their work, they prove that geopolitical events and rare-earth raw material prices are tightly connected, and this connection is bidirectional: higher prices increase geopolitical risk, while geopolitical tensions affect prices also. The authors also investigated the effect of global industrial activity on rare-earth raw material prices, and they documented the expected positive connection between these factors. There is increasing literature on the subject since the observed increase in rare-earth permanent magnet market is demand-driven [28,29,30,31].

Carefully targeted substitution can reduce the cost of a compound and improve the magnetic properties as well as the energy product. Much research has been carried out in the last few years to deal with these drawbacks. New intermetallic compounds based on SmCo_5_ suitable for permanent magnets are produced by substitution of Fe, Ni, Cu or other transition (or non-transition) elements for Co or by reducing the content of expensive raw rare-earth materials with other rare-earth atoms, which may present larger abundance worldwide or less demand for applications and thus include less criticality and supply risk, like Ce and La. Ab initio methods like DFT-based calculations and other theoretical methods are also widely used to clarify and predict if it is possible to follow the two routes mentioned above [13,17,18]. High-entropy alloys are another interesting focus of research; however, reports on such alloys will not be included in our study. This review aims to present the progress of recent years in the efficient replacement of high-cost elements in SmCo_5_-type magnets.

## 2. A Short View of Structure and Magnetic Properties of SmCo_5_ Magnets

**Figure 1:**
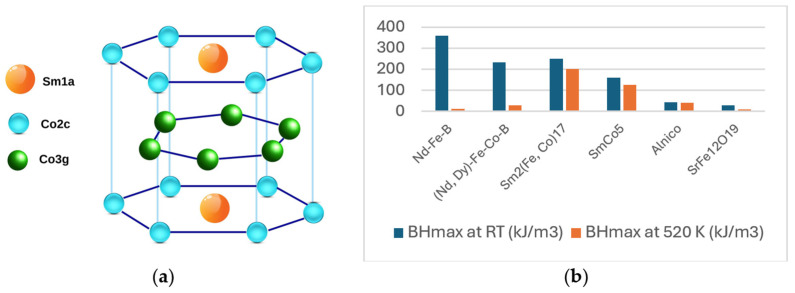
(**a**) Crystal structure of SmCo_5_. (**b**) Energy products of classes of permanent magnets.

RCo_5_ (R = Sm, Nd, Y, Ce) permanent magnets have been reported since 1967 [6]. The development of these magnets started with the SmCo_5_ compound and are made from an alloy consisting of samarium and cobalt, often combined with traces of other rare-earth elements such as praseodymium and neodymium. SmCo_5_ compounds crystallize in the hexagonal CaCu_5_-type structure with three non-equivalent atomic sites: Sm1-(1a), Co1-(2c), Co2-(3g) [32,33]. The crystal structure of SmCo_5_ is shown in Figure 1a.

SmCo_5_ exhibits relatively high saturation magnetization (M_s_) and at the same time a good temperature endurance with a Curie temperature (T_c_) up to 1020 K and large magnetocrystalline anisotropy constant K_1_ of about 17.2 MJ/m^3^ [34,35,36]. The combination of these properties produces large coercivity, and its origin may be traced to the rare-earth sublattice, whereas the transition metal sublattice contributes to the hard magnetic properties and stabilizes via intercalative exchange the anisotropy at elevated temperatures. Some magnetic properties of SmCo_5_ and other compounds are given in Table 1. It can be observed that Nd_2_Fe_14_B has an energy product of 64.34 MGOe at RT, which drops to 1.52 MGOe at 523 K, while SmCo_5_ has an energy product of 20 MGOe, a third that of Nd_2_Fe_14_B at RT, but much higher at 523 K [17,35,36].

## 3. SmCo_5_ Transition Metal Substitution (Fe, Cu, Ni, Zr, Ti, and Nb)

The group of transition metals are sometimes mentioned as the d-block elements due to the fact that d electrons are the external unfilled shells. They are contained within the middle area of the periodic table and are most important for magnetic materials belonging in the fourth period (Sc, Ti, V, Cr, Mn, Fe, Co, Ni, Cu, Zn), with the exception of Nb, which belongs to the fifth. The electron configuration in is (*n*−1)*d*^1–10^ *ns*^2^; however, in some cases in intermetallics, the electrons are distributed in a different manner and subshells or partially filled orbitals may arise. Most common cations have a valence of +2 or +3, but there are some that may provide only one electron forming +1 cations or in some cases higher.

### 3.1. Substitution of Fe for Co

SmFe_5_ is a metastable compound and is not present in the Sm–Fe phase diagram. The initial focus on partially replacing Co with Fe was due to the obvious fact that Fe is more abundant and thus cheaper. Fe is the second-most abundant metal in the Earth’s crust after aluminum. This could reduce the price of raw materials needed for applications and potentially improve magnetization. Equilibrium solubility of Fe in SmCo system is limited, making this specific substitution difficult. The formation of metastable Sm (TM)_5_ (TM = Fe-Co) compounds and their magnetic properties has been reported on by Miyazaki et al. since 1988 [37,38]. In that work, the rapidly quenched Sm (Co_x_Fe_1−x_)_5_ alloy ribbons were investigated. These ribbons were prepared at a velocity of 41.9 m/s and found to exhibit amorphous microstructure in the stoichiometric range 0 ≤ x ≤ 0.2 for Sm (Fe_1−x_Co_x_)_5_.

It was depicted that the structure is retained in the range 0.6 ≤ x ≤ 1.0 and single-phase alloys were produced by quenching. The single phase Sm(TM)_5_ compound exhibits large coercivity of about 5 to 14 kOe. Despite large coercivity values, the performance of the materials was hindered by the low values of magnetization, which were measured at 16 kOe, lower than expected. It is well known that Fe has the highest atomic magnetic moment, but the coercivity of the ribbons with more 50% replacement of Co for Fe decreased.

**Figure 2:**
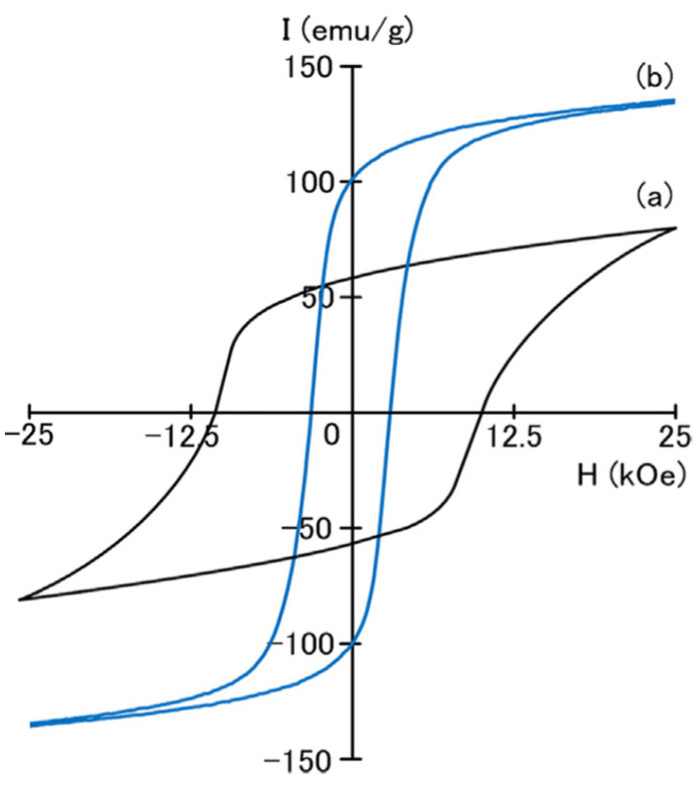
Hysteresis loops of Sm–Co–Fe melt-spun ribbons annealed at 873 K: (**a**) SmCo_4_Fe and (**b**) SmCo_2_Fe_3_ alloys. Reprinted from [39] with permission from Elsevier.

Similar works to those mentioned above are also reported in [39,40], where the investigation of structural and magnetic properties of alloys with SmCo_5−x_Fe_x_ (*x* = 0–4) stoichiometry produced in the form of ribbons by melt-spinning. XRD and thermomagnetic studies revealed that the hexagonal Sm(Co, Fe)_5_ phase can be retained with replacement up to *x* = 2 and that the large substitution of Fe for Co in the alloys will produce the appearance of other intermetallic phases like Sm(Co, Fe)_7_ and Sm_2_(Co, Fe)_7_. Heat treatment of the materials increases the remanence up to *x* = 3, and further increase in Fe content will reduce both coercivity and remanence. This heavy dependence of structural and magnetic properties in the system on Fe content is the common ground in all similar studies. Annealed SmCo_4_Fe alloys exhibited a relatively high coercivity of 10.2 kOe combined with a remanence of 60 Am/kg (Figure 2), while thermally treated SmCo_2_Fe_3_ alloys were utilized in a nanocomposite magnet with a high remanence of 100 emu/g, but a rather low coercivity of 2.9 kOe [39].

Although these results did not encourage the intensification of studying substitution of Co only by Fe, the efforts continued with the addition of theoretical tools [40,41,42]. Most of the findings are consistent with previous research. The origin of magnetocrystalline anisotropy and the effect of Si was described in [41]. In this case, the substitution scheme included both magnetic and non-magnetic atoms (Fe/Si). The magnetic hardness is mostly due to the atoms in 2c positions, the layers that also contain the rare-earth atoms. The Sm 4f electrons that belong to the highly asymmetric and localized orbital play a vital role. When Co atoms are replaced in 2c positions, the 4f orbitals are broadened and shifted towards the Fermi level, and this has a negative effect on the anisotropy. In the other layer of the structure, which is occupied by Co in 3g positions, the main contribution to the total magnetization can be traced; the presence of Fe atoms in these positions increases the magnetic moment to 14.02 μ_B_ in total and has a positive effect in the anisotropy. Si substitution favors thermodynamic stability in high-Fe content, but has a negative effect on the magnetic properties, both magnetization and anisotropy. Recent theoretical studies of Sm(Co_1−x_Fe_x_)_5_ are in agreement with the experimental results we have described [42]. According to these calculations, magnetization is expected to increase up to 10.6 μ_B_ from 7.8 μ_B_ by substitution of Co for Fe up to 80%. A small preference of Fe atoms for 3g crystallographic site was suggested and a Slater–Pauling-like behavior depending on the Fe content was calculated.

### 3.2. Substitution of Cu and Other Transition or Non-Transition Elements for Co on SmCo_5_ Alloys

The replacement of cobalt with copper was also reported in several studies in the early eighties. In some cases, they result in solid materials with significant permanent magnet properties. In magnetic materials with R(Co, Cu)_5_ stoichiometry, large coercivity values have already been reported in alloys in the as-cast form; these properties can be further enhanced by thermal treatment. Coercive forces in the vicinity of 28–30 kOe have been obtained in annealed samples [42,43]. Some research on SmCo_1−x_Cu_x_, as cast, annealed alloys, or single crystal, was published in the 1970s and 1980s [44,45,46,47,48,49,50].

According to Oesterreicher et al. [49] the magnetic hardness in pseudo-binaries like SmCo_5−x_Cu_x_ may already be an intrinsic property in nature and does not dependent on mechanisms such as pinning by other grain boundary phases. Under this scheme, coercivity is affected by temperature following a typical model based on thermally driven propagation of domain walls.

Metallographic and microprobe analysis of SmCo_1−x_Cu_x_ indicates the presence of spinodal decomposition in the metastable as-cast material [44,45,46]. This relates to the increase in coercivity after heat treatment in Sm(Co,Cu)_5_ alloys, even under relatively low temperatures in the area of 300–500 °C; this has been correlated with spinodal decomposition into both Co- and Cu-rich Sm(Co,Cu)_5_ phases [44,46]. Curie temperature determination and further DTA analysis have been depicted in a hypothetical Sm–Co–Cu ternary phase diagram. The critical initial amount of copper leads to decomposition within the range 24 to 40 at. % [46]. Resulting components retain the hexagonal CaCu_5_-type structure but exhibit a disorder in the composition concerning the transition metal. Subsequent annealing at elevated temperatures in the area of 1073–1273 K settles the stoichiometry of the materials. It seems that annealing affects the coercivity mechanism. The dominant domain-wall nucleation in the as-cast samples with relatively weak pinning at the grain boundaries changes towards a stronger domain-wall pinning process in the annealed alloys. Electron microscopy analysis of as-cast samples with moderate Cu content provided evidence for the existence of three phases: two of them retained the hexagonal CaCu_5_-type structure but were Co-rich and Cu-rich, and an intergranular Cu-rich Ce_5_Co_19_-type phase [47,48].

**Figure 3:**
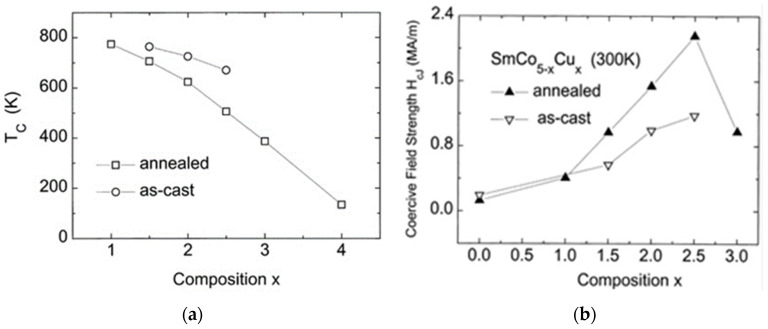
(**a**) Dependence of the Curie temperature on the concentration of SmCo_5−x_Cu_x_ compounds. (**b**) Coercive force at room temperature as a function of the chemical composition of Sm-Co-Cu alloys in the as-cast state and after heat treatment at 1273 K for three weeks and subsequent quenching. Reprinted from [51] with permission from Elsevier.

Later in the 1990s, the properties of Sm(Co,Cu)_5_ magnets were systematically studied over the full stoichiometry range and in a large temperature area, as reported by Blanco et al. [39,40,41,42,43,44,45,46,47,48,49,50,51]. They prepared polycrystalline SmCo_5−x_Cu_x_ (*x* = 1, 1.5, 2, 2.5, 3, 4) samples, both as cast and thermally treated. They observed that Curie temperature weakens with increasing Cu content (Figure 3), reaching very low values for 80% replacement of Co by Cu (Figure 3a). Coercivity presents intense temperature and stoichiometry dependence and reaches a maximum for SmCo_2.5_Co_2.5_ ribbons (Figure 3b). It seems that Cu atoms act like local defects enhancing the coercive field. In particular, they also measured the hysteresis loops in SmCo_5−x_Cu_x_ as-cast and annealed magnets (1 < *x* ≤ 3) at room temperature using a pulsed-field magnetometer and a static vibrating sample magnetometer and noticed a giant magnetic viscosity effect, proportional to copper content [52].

The internal mechanism that produces the large coercive fields in the Sm(Co_1−x_Cu_x_)_5_ (0 < x ≤ 1) compounds has been also studied by focusing on structural parameters, namely, the coherence between unit cell parameters of Sm(Co, Cu)_5_ and hcp Co with coercivity [53]. In this work, it was suggested that cobalt precipitates along grain boundaries may affect the coercivity in Sm(Co,Cu)_5_ alloys. It was also mapped that intrinsic coercivity is optimized within the area between 60% and 80% replacement of Co by Cu. In all these cases, elevated thermal treatment was applied. Tellez-Blanco et al. annealed Sm(Co, Cu)_5_ samples for 504 h (21 days) at 1000 °C [51,52]. Nishida et al. used a lower annealing temperature above 800 °C for 160 h [54]. Gabay et al. used a more complex approach: thermal treatment of the Sm(Co, Cu)_5_ magnets for 100 h in two steps. The first lasted 50 h at 1050 °C and then another 50 h in a temperature range of 350–450 °C [55]. As such, the synthesis of Sm–Co–Cu ternary alloys must be considered a high-energy-consumption process.

**Figure 4:**
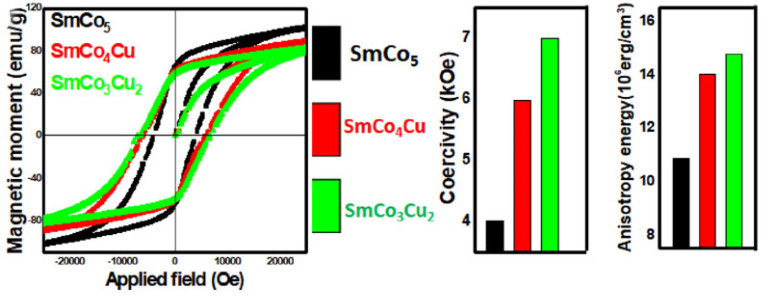
Magnetic hysteresis loops of SmCo_5_ and SmCo_5−x_Cu_x_. Variations in magnetic moment, Hc, and anisotropy energy, before and after Cu substitution, obtained from [56], which is published as an open-access article and distributed under the terms of the Creative Commons CC BY license.

A more recent work deals with these problems using reduction diffusion to synthesis, with improved energy and time efficiency compared to the previous metallurgical methods [56]. In this study, Haider et al. applied an optimized chemical method that is more energy-efficient for the synthesis of Cu-substituted SmCo_5_ compounds. Following this scheme, chemical precursors including samarium, cobalt, copper and potassium chlorides (SmCl_3_, CoCl_2_, CuCl_2_, KCl) were utilized for the synthesis of Sm(Co,Cu)_5_ particles by adjusting the molar ratios of the reagents in order to keep Cu introduction in moderate amounts. Final products were thermally treated at 900 °C for only 2 h, a much shorter annealing time than traditional approaches, reducing overall preparation cost. Cu introduction in 2c positions in the hexagonal SmCo_5_ crystal structure leads to weakening of the coupling in the surroundings. The resultant decoupling obviously affects all the magnetic properties of moment, magnetocrystalline anisotropy and coercive field (Figure 4). The magnetic moment was profoundly reduced, but coercivity and anisotropy were enhanced as a result of Cu substitution for Co. Enhancement of the magnetocrystalline anisotropy energy also affected the coercivity positively, and values of 4.50, 5.97 and 6.99 kOe for SmCo_5_, SmCo_4_Cu and SmCo_3_Cu_2_, respectively, were measured (Figure 4).

**Figure 5:**
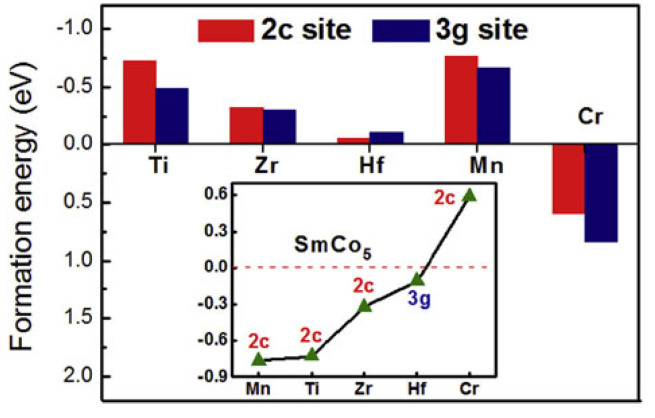
Formation energy of the doping element at 2c and 3g sites. The insert shows the formation energy of SmCo_4.5_M_0.5_ with different elements at the preferential site. Reprinted from [58] with permission from Elsevier.

In addition to Fe and Cu substitution materials for Co in SmCo_5_, alloys with substitution of other elements, such as Ni, Pt, Cu, Ag, Al, In, Si and Sn (transition or non-transition elements), have been synthesized [57]. The enthalpy of formation for some doping elements when they are introduced in 2c or 3g positions of alloys with nominal SmCo_4.5_M_0.5_ stoichiometry are shown in Figure 5. Magnetic properties have been investigated at liquid helium temperature. In general, substitution of Co by non-magnetic elements like Al and Si weakens magnetic properties, especially saturation magnetization; however, when Al and Si replace Co in SmCo_5_, the result is an outstanding increase in coercivity at values in the range of 30–50 kOe, even in bulk materials. Most of the other chemical combinations yielded unsatisfactory results.

The origin of these hard magnetic properties is usually discussed in terms of domain-wall pinning by atomic dimension centers that act as obstacles. Additionally, first-principle calculations and statistical thermodynamics can be utilized for studying the composition-dependent structural stability and the relevant magnetic and electronic properties of alloys with mediocre substitution of Co by other transition metal elements, e.g., alloys with nominal composition of SmCo_4.5_M_0.5_, M = Ti, Zr, Hf, Mn and Cr [58]. The addition of Ti, Zr, Hf and Mn was beneficial for the stability of the SmCo_5_ phase, while doping of Cr was detrimental. It was found that the SmCo_4.5_M_0.5_ ternary alloys can be stabilized by Mn, Ti and Hf introduction over an even wider temperature range, while the overall magnetic moment of the SmCo_4.5_M_0.5_ system was usually weakened by non-magnetic element doping, as expected. On the contrary, doping with Mn increased the total magnetic moment.

### 3.3. Simultaneous Substitution of Two or More Transition Metals Like Fe and Cu for Co in SmCo_5_ Alloys

Gabay et al. studied the possibility of stabilizing compounds with nominal composition RCo_5−*x*_Cu*_x_* (R = Y, Sm) with respect to phase separation [55]. They utilized first-principle density functional calculations, and their observations imply that decomposition of the material into two separate phases with different copper content is energetically favorable. They also estimated that the magnetic state of the alloys is connected to parameters like the Cu content, which can be accommodated, and Cu atomic position preference. Thermal treatment of samples of alloys with nominal compositions of SmCo_4_Cu_1_, SmCo_3.5_Cu_1.5_, SmCo_3_Cu_2_ and SmCo_2.25_Fe_0.75_Cu_2_ affects the application of important magnetic properties, notably the Curie temperature and coercivity. The latter increases significantly if the annealing temperature is 100–140 °C below the Curie temperature. For example, in the case of SmCo_2.25_Fe_0.75_Cu_2_, room-temperature coercivity increases from 12.3 kOe to 37.3 kOe. A difference from other studies, especially [43,44,45], these experimental results do not seem compatible with the theory of spinodal decomposition. Another possibility suggested is that the improvement in coercive force has to do with differences in the occupancies of the transition metal atomic positions.

Concerning the maximum energy product of the final magnets based on the SmCo_5_ alloy, the main limiting factor is the remanent magnetization of the basic ingredient. Exploration of both Fe- and Cu-substituted alloys has been successfully applied in a SmCo_5_ system [59]. Additionally, partial substitution of Sm by Zr has been applied, as in the case of the related Sm_2_Co_17_ magnets: Zr can replace both transition metals and rare earths in relevant intermetallics [60]. Cu accumulates in the grain boundary phase and is responsible for the enhancement of coercivity under the domain-wall pinning mechanism [61]. The magnetic properties of these kinds of as-cast alloys depend strongly on the annealing temperature and cooling rate. The best prepared magnet with composition of SmFe_0.4_Co_3.5_Cu_1.1_ has a maximum energy product of 13 MGOe. This alloy was annealed for 2 h at 1100 °C, then slowly cooled in argon atmosphere [62]. Initial magnetization curves of as-spun SmCo_3.839_Cu_0.48_Fe_0.48_ ribbons and the variation in magnetization versus wheel speed at 20 kOe were generated [62]. Ribbons produced with higher wheel speed (≥30 m/s) exhibited single-phase SmCo_5_-type structure. At higher wheel speed, the results were even more encouraging: a large value of coercivity of about 33 kOe was obtained in ribbons prepared at 50 m/s. This was attributed to the formation of single-phase Sm(CoCuFe)_5_ crystallites, and in optimization of their size, grains of the high anisotropic phase are smaller due to the more intense cooling introduced by higher wheel speed.

It is worth mentioning the work of Zhu et al., which focused on the substitution of both Cu and Ti for Co Sm-Co magnets [63], a new class of materials with promising potential for usage in permanent magnet applications. They achieved an appreciable high-temperature coercivity of 8.6 kOe at 500 °C. Thermomagnetic analysis and temperature-varied X-ray diffraction patterns revealed that their samples were two-phase mixtures of the crystallographically related 2:17 and 1:5 type structures, and their combination was the reason for these interesting properties.

## 4. Substitution of p-Elements for Co

**Figure 6:**
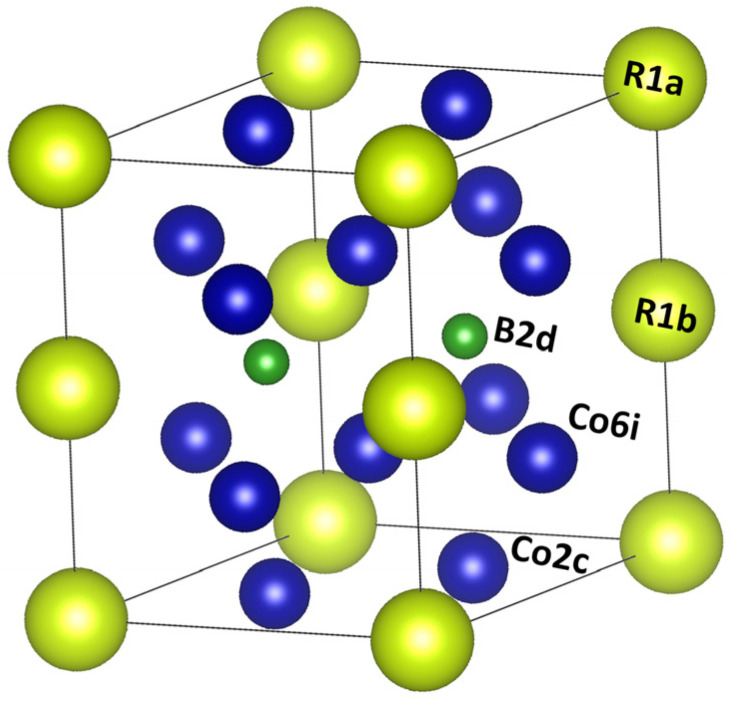
Crystal structure of the *R*Co_4_B compounds (green spheres represent R atoms in 1b sites; blue spheres the atoms of Co in 3g sites and B represents B atoms in 2d sites) [69].

Another possible improvement is the partial introduction of p elements like Al, B, Ga instead of Co in the RCo_5_ (R = Y, Pr, Nd, Sm, Gd, Tb) alloys [64,65,66,67]. In [64], the crystallographic and the magnetic properties of alloys with nominal composition RCo_4_B (R = Y, Pr, Nd, Sm, Gd, Tb) are presented. The RCo_4_B compounds crystallize in a hexagonal CeCo_4_B-type hexagonal structure (S.G. P6/mmm); this phase is a derivative of the basic RCo_5_-type structure [68] and is produced by a regular substitution of Co atoms by B ones in every second layer of the CaCu_5_ structure parallel to the basal plane. A description of the structure is depicted in Figure 6. It shows that the R atoms now occupy two distinct sites, denoted 1a and 1b, Co atoms are distributed in the 2c and 6i positions, and the B atoms reside in the 2d positions. The saturation magnetization M_s_ and the Curie temperature T_c_ were found to reduce upon the B for Co substitution. However, the SmCo_4_B compound still possessed good overall magnetic properties and had reduced Co content, thus making it a promising candidate for permanent magnet applications.

An interesting observation is the effect on the unit cell parameters of different rare-earth atoms. It is well known that with increasing atomic number across the lanthanide series, the ionic radius reduces (lanthanide contraction). In the case of the RCo_4_B family, the c-axis of the unit cell of the hexagonal structure remains almost constant, while the a-axis slightly follows a rare-earth atomic radius, increasing when the atomic number is reduced. The alloys with R = Y, Pr, Nd are ferromagnetically ordered; however, heavy rare-earth atoms and Gd produce ferrimagnetic compounds. Easy magnetization axes at ambient temperatures depend on R content. In contrast to the substitution effect of B for Co in the RCo_5_ compound, which crystallizes into a CeCo_4_B type of structure, the substitution of Co by Ga and Al preserves the CaCu_5_-type structure of RCo_5_ [70]. Some magnetic properties like the saturation magnetization and the Curie temperature are weakened when Ga or Al replace Co; however, the magnetocrystalline anisotropy and subsequently the coercive field are stronger in a similar manner to the Cu introduction.

As mentioned above, introducing Cu or Al to SmCo_5_ positively affects coercivity, but negatively affects magnetization. A question arises as to whether a multiple substitution, for example, Al, Cu and Fe for Co, could improve both the stability and the magnetic properties of the compounds. This has been the scope of works like those in [71,72]. Samples of alloys with nominal composition SmCo_5_ + xAl_82.8_Cu_17_Fe_0.2_ (*x* = 3–7) in the form of ribbons were melt-spun at a wheel speed of 40 m/s. Glassy in the as-span form, subsequent thermal treatment improved the crystallinity and introduced changes in the phase balance and the microstructure of the materials. The 3–5 wt% Al-Cu-Fe alloying addition was found to be the optimum for improving the desired magnetic properties. Annealing the alloys at temperatures about 500 °C increased both the coercivity and mass magnetization. Even at elevated temperatures up to 600 °C, these materials presented a large coercivity of 26.8 kOe, but with the usual trade-off in saturation magnetization. The increase in coercivity at elevated temperatures was attributed to a large amount of Sm(Co, M)_5_ grains, which improved the crystallinity of the material and the size optimization of the grains.

## 5. A New Promising Magnetic Material with CaCu_5_ Structure

**Figure 7:**
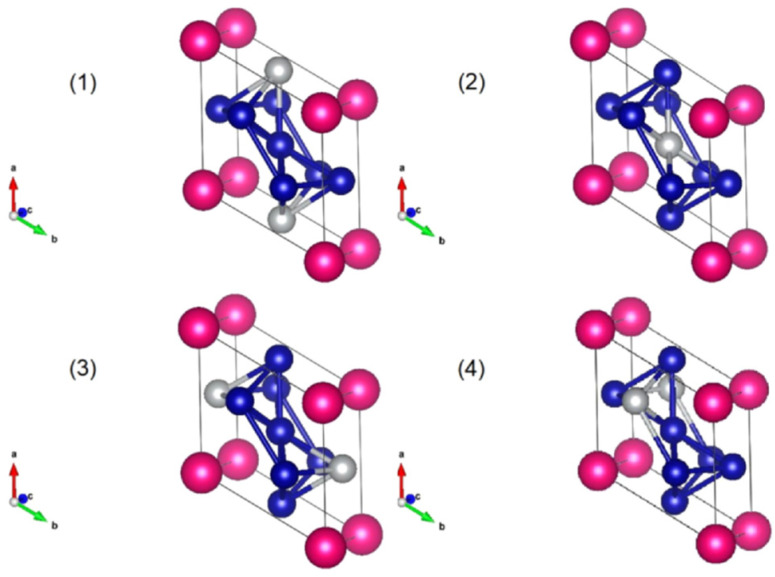
Schematic illustration of four different cases for the arrangement of Co and Ni atoms in the unit cell of SmCo_4_Ni. Cases **1**, **2** and **3** are degenerated, Case **4** can be produced by symmetry operations. Red spheres represent the atoms of Sm, blue spheres the atoms of Co and silver spheres the atoms of Ni. Reprinted from [73] with permission from Elsevier.

A new boost in the research efforts in the SmCo_5_ family of alloys was initiated by ab initio and other computational methods. The effect of partial substitution of Co by Ni on the crystal structure of the basic SmCo_5_ compound has been investigated by computational methods [73,74,75,76,77,78,79] with the aim of determining the composition that will optimally stabilize the structure and maintain good magnetic properties at the same time. Ab initio atomistic simulations implemented using density functional theory calculations are a useful tool for determining the stability and other properties of intermetallic compounds. For example, in the case of the SmCo_5−x_Ni_x_ series (*x* = 1–5), all possible crystallographic combinations of Co and Ni atoms in the hexagonal structure can be tried and relevant formation energies calculated [73]. A schematic illustration of four different cases of the unit cell of SmCoNi_4_ is shown in Figure 7.

In this work, an experimental implementation based on 20% Ni replacement for Co was presented in order to correlate the findings from calculations with realizable bulk materials. A very interesting observation was that sometimes the atomic configuration that leads the system to the minimum energy is not the one that produces the maximum magnetization. In the case of the experimentally investigated SmCo_4_Ni stoichiometry, this is depicted since both the least energy and the atomic arrangement that maximizes the magnetization have been identified [73]. As depicted in previous results, introduction of Ni in the SmCo_5_ system does not favor maximum magnetization. Ni is also an expensive raw material, as already discussed above. Considering the cost and criticality, introduction of Fe is a preferable objective. Fe is about 2000 times more abundant than Co in Earth’s crust and consequently cheaper and less critical. Fe as a metal possesses the largest magnetization at room temperature (1.76 MA/m) [60]. SmFe_5_ (full substitution of Co for Fe) is thermodynamically unstable and does not exist. However, binary Sm-Fe alloys belonging in the SmFe_5_ phase were produced for the first time in the form of ribbons by utilizing rapid quenching and carefully optimizing the annealing parameters [74]. SmFe_5_ grains were detected in the ribbons, and after thermal treatment at 1073 K, the samples presented a coercivity of 1.2 kOe.

This issue (only replacing Ni for Co decreases the T_c_ and M_s_ and only replacing Fe for Co makes the system unstable) was recently addressed by two theoretical studies, where the stability of modified SmCo_5_ alloys by partial replacement of Co by Fe and Ni at the same time was investigated [75,76]. Based on ab initio calculations, Söderlind et al. suggested a very efficient permanent magnet with nominal SmCoNiFe_3_ stoichiometry, which was thermodynamically stable while crystallizing in the well-known SmCo_5_ type. They observed a connection between the number of transition-metal 3d electrons and the subsequent stability of the hexagonal structure of the SmM_5_ compound (M = Fe, Co, or Ni). The dependence of the experimental energy of formation on the number of 3d electrons was plotted [75]. The calculated formation energy of SmCoNiFe_3_ is on the negative part of this extrapolated curve, indicating a thermodynamic stability of this hypothetical compound. The values of energy formation for SmM_5_ (M = Ni, Co) were taken from [77,78].

By means of first-principle electronic structure calculations, they found that the magnetic properties of SmCoNiFe_3_ (energy product, T_c_, Ms) are close to the respective properties of Nd-Fe-B types of magnets (Table 2). Substituting most of the Co with Fe in SmCo_5_ and doping with a small amount of Ni could be a path towards a new material for permanent magnet applications, which could combine adequate values for all critical magnetic properties and could potentially interchange SmCo_5_ or even Nd-Fe-B based magnets in several applications.

The enthalpy formation energy of SmCoNiFe_3_ was just below the dividing line of the stable–unstable enthalpy formation region. Increasing the number of 3d electrons, i.e., increasing the Ni content, has a positive effect on the stabilization of the compound. It is underlined that an alloy in this family of compounds is stable when it incorporates at least 7.2 3d electrons per transition metal atom. For SmCoNiFe_3_, this value is 7.3, which is marginal but probably enough to stabilize the compound.

Despite the confidence in the manufacturing of SmCoNiFe_3_, the experimental synthesis of this compound has not yet been reported. An approach toward this synthase was attempted by Gavrikov et al. and G. Sempros et al. [79,80]. Based on calculations, ribbons with nominal composition Sm(Co_1_*_−__x__−__y_*Fe*_x_*Ni*_y_*)_5_ (*x* = 0.15, 0.3, 0.45; *y* = 0.05, 0.1, 0.15) were prepared [81]. The ribbons consisted of two phases, the main phase being SmCo_5_ (up to 70 wt%), while the other belonged to Sm(CoFe)_3_-type. Co reduction points towards lattice expansion, decrease in Curie temperature and coercivity and improvement of mass magnetization. The maximum Ni and Fe content was in a sample with SmCo_2_Fe_2.25_Ni_0.75_ stoichiometry. Among their samples, SmCo_3_Fe_1.5_Ni_0.5_ presented the highest coercivity at about 10.9 kOe and the largest remanent magnetization of 51 Am^2^/kg, the highest energy product.

According to the predictions of ab initio simulations [80], the SmCo_2.5_Fe_1.5_Ni composition presents a large mass magnetization, not much smaller than the value of the mother SmCo_5_ compound. By simultaneously substituting Co with Fe and Ni, a favorable interplay between stability and magnetization could be tuned, benefiting from the additional advantage of the reduction of Co content. However, the experimental values for the critical magnetic properties—especially mass magnetization—of SmCo_2.5_Fe_1.5_Ni were found to be lower than predicted, possibly due to secondary impurity phases.

## 6. Recent Experimental and Theoretical Research Approaches on SmCo_5_ Alloys: The Case for Mm (Misch-Metal) Substitution for Sm on SmCo_5_ Alloys

Replacing Co with other transition or non-transition elements improves magnetic properties such as coercivity and anisotropy field at the expense of others such as energy product or does not produce stable phases, suitable for bulk permanent magnets [74]. Thus, a possible substitution scheme in the SmCo_5_ alloys cannot rely entirely on a transition metal (or non-transition element) sublattice but could also include that of rare earths. Sm replacement could be realized with a light rare-earth atom with an atomic number less than Gd. These atoms have negative Stevens’s coefficients, as opposed to Sm, which has a positive one [5]. This practically means that their 4f orbital has a different shape and possibly favors different arrangements within the crystal field of the material, making it possible that they could provide different magnetic properties in the case of simultaneous substitution within both the rare-earth and transition metal sublattices. Within the lanthanide series, there are also variations in the relative content of the reserves. Most of the mining sites around the world are composed of minerals that contain a mixture of various rare-earth oxides, but their relative amounts are not the same. Heavy rare earths are less abundant, while some elements, namely, Ce and La, are practically overproduced [81], sometimes referred as “free rare earths”. This fact is often denoted as the “rare-earth balance issue” [82].

A high-performing permanent magnet seems unlikely to be manufactured with Ce or La as a basic ingredient, due to the specific properties of these atoms and especially their electronic configuration. However, a partial replacement of an expensive rare-earth element by a less expensive one may establish a material that could be used as the basis for a “gap” magnet [15] and may find potential use in applications with low cost. Due to the different shape of the 4f orbital and thus the impact on the nature of the interactions within the crystal field, this partial replacement may provide a path towards replacement of high-cost Sm in the Sm-Co system.

Development of new materials and alloys using selective La-Ce modification has been demonstrated recently in the case of Nd-Fe-B-based permanent magnets [83,84]. The introduction of Ce and La in the SmCo_5_ system has attracted attention as early as the discovery of the initial compound. M. G. Benz and D. L. Martin studied Sm-Co magnets, where Sm was partially substituted by misch metal [85]. In the specific study, the misch metal contained a large amount of other rare earths, mostly Nd, but three quarters of the mass content were Ce and La in a 2:1 mass ratio. Results were encouraging, since a BH_max_ of about 20 MGOe was obtained, about 60% of SmCo_5_-based permanent magnets, while possessing such advantages as simplification of overall processing and compatibility with existing industrial infrastructure. Some magnetic properties are improved, and regarding the supply cost, the overall merit of the material is improved. Another important research area towards development of new materials suitable for applications as permanent magnets is the theoretical understanding of the fundamental underlying physics.

Lately, a recent series of theoretical as well as experimental studies developed the possibility of simultaneously introducing metallic Ce-La at a Ce:La ratio of 3:1 into the SmCo_5_ system and reducing the Co content [80,86,87,88]. The theoretical framework of the compounds and the relevant ab initio parameters were intensively investigated. It was also detected that the Ce electronic states are located closer to the Fermi level compared to the other cases [87]. This is an indication of the important role of Ce atoms in the modification of the magnetic properties upon substitution of Sm. However, the most dominant factor for magnetization is still the Co–Co interatomic distances.

**Figure 8:**
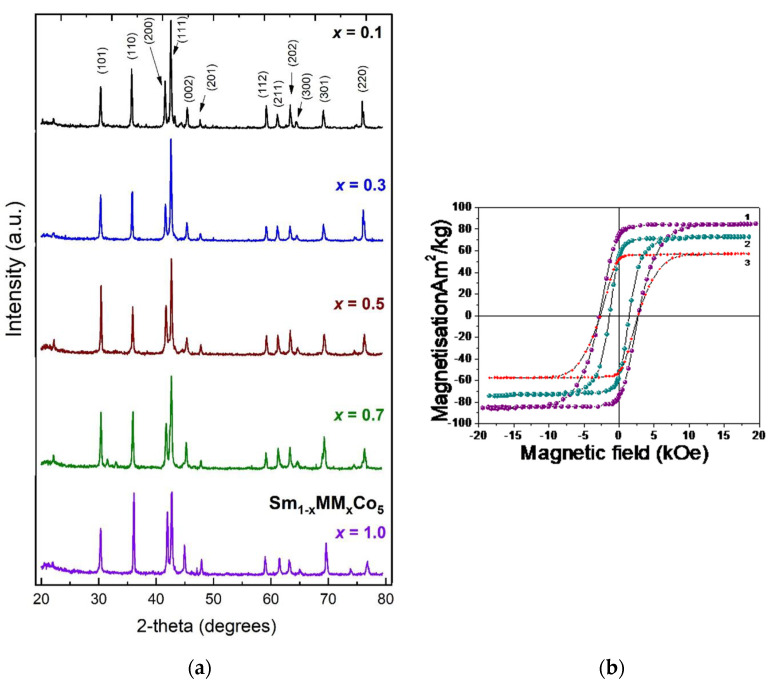
(**a**) X-ray diffraction patterns (Cu Kα radiation) of Sm_1−x_Mm_x_Co_5_ samples [86]. (**b**) RT hysteresis loops of Sm_0.5_Mm_0.5_Co_4_Fe_0.5_Ni_0.5_ (1) Sm_0.5_Mm_0.5_Co_3_FeNi (2) and Sm_0.5_Mm_0.5_Co_4_Ni (3) samples [87].

Sm_0.5_MM_0.5_Co_5_ was also synthesized experimentally yielding a relatively high Curie temperature of 828 K and fair saturation magnetization of about 61 Am^2^/kg. X-ray diffraction patterns (Cu Kα radiation) of Sm_1−x_MM_x_Co_5_ samples and RT hysteresis loops of Sm_0.5_MM_0.5_(CoFeNi)_5_ are shown in Figure 8. The whole range of synthesized (SmMM)Co_5_ alloys exhibit uniaxial magnetocrystalline anisotropy and hard magnetic properties. The experimental value for magnetization is lower than theoretical calculations, a somewhat expected result due to minor defects in samples. The feasibility of misch metal introduction in the SmCo_5_ system was confirmed both experimentally and theoretically. Considering that MM is much cheaper than Sm, this material could serve as a possible candidate for applications in the “gap” performance region we described earlier.

**Figure 9:**
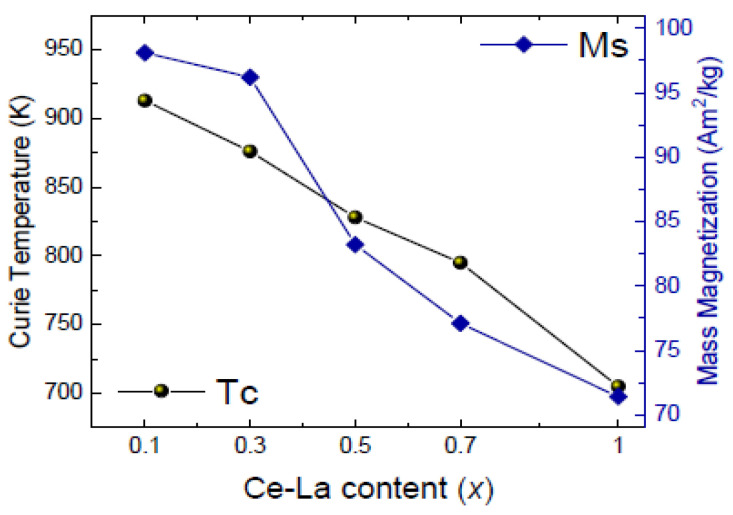
Dependence of magnetic properties on Ce, La content in Sm_1−x_(Ce_0.75_La_0.25_)_x_Co_5_ (x = 0.1–1.0) samples [86].

Curie temperature and mass magnetization both drop about 30% from SmCo_5_ to MMCo_5_, almost linearly, with the compounds with 30% substitution still presenting almost all the SmCo_5_ basic compound’s potential. Curie temperature of Sm_0.7_(Ce-La)_0.3_Co_5_ was measured at 876 K and mass magnetization at 96 Am^2^/kg (Figure 9). For high Ce_3_La content. the Th_2_Ni_17_-type phase is also found. Due to their large crystallographic similarity, the latter can be considered a derivative of the original CaCu_5_-type structure by replacing a third of the rare-earth atoms with pairs of transition metal atoms, here Co, arranged along the c-axis.

These pairs are called dumbbells. All SmCo_5_ derivatives must satisfy Stadelmaier’s criterion—one crystallographic unit cell must obey the equation 𝑝_der_ ≈ _(1:5)_∙√3—in this case, the a-axis on the basal hexagonal plane [89]. Ab initio theoretical calculations confirmed the most important results, with the exception of magnetocrystalline anisotropy energy. Magnetocrystalline anisotropy energy cannot be predicted correctly in conjunction with atomic magnetic moments using the same potential within relativistic linearized augmented plane wave (FLAPW) approach. P. Larson et al. [88] pioneered the discussion of this issue by using an extended model containing a potential U beyond the local density approximation (LDA + U); this potential adjusts the electrostatic coulomb interaction of the rare-earth f orbital [88]. Exchange potentials are corrected with generalized gradient approximation (GGA) [89]. In their work, they followed an approach typical for experimental investigation of anisotropy in rare-earth transition metal intermetallics: they studied, theoretically, the Y member of the family, YCo_5_ in this case, where they detected that the Co atom layout contributes to magnetocrystalline anisotropy energy, a minor contribution, but larger than pure Co hcp arrangement. The spin-down density of states is observed to present a peak close to the Fermi energy. Of course, the main source of anisotropy in the material are the Sm 4f electrons, which are spin-orbit coupled, and their highly asymmetric orbital interacts with crystal field, as mentioned already. Treating those electrons as localized rather than “open core” and a selection of U above 5 eV predicts magnetocrystalline anisotropy energy close to the experimental values (10–15 meV), but leads to a discrepancy of more than 2 Bohr magnetons in atomic magnetic moment. On the other hand, lower selection for U in the vicinity of 4 meV accurately predicts the atomic magnetic moments while significantly overcalculating magnetocrystalline anisotropy energy. This is in general the common ground among many works in literature [75,76,86,88,89,90,91,92,93,94,95,96,97]. J. X. Zhu et al. have combined LDA with Dynamic Mean Field Theory (LDA + DMFT) to investigate the correlated electron effects on the magnetocrystalline anisotropy energy, since they play a crucial role to orbital component of atomic moments and consequently those moments directly affect the magnetocrystalline anisotropy. Their main assumption is that both orbital and spin factors of atomic moments originate from the correlated shells, and they structured accordingly Green and Weiss functions in their calculations. The inclusion of DMFT results in a higher estimation of the orbital moment. Their calculated magnetocrystalline anisotropy energy of YCo_5_ of 3.5 meV/f.u. is consistent with experiments (3.2 meV/f.u.). Under this approach, the Coulomb potential U remains important as well as the spin orbit coupling, but the importance of correlated systems is underlined [95].

C. Patrick and J. Staunton [97] proposed using self-interaction-corrected DFT (SIC-DFT), an approach that removes the self-interaction error that hinders the performance of simple DFT calculations. They also employed the disordered local moment (DLM) picture of magnetism in order to adjust for the effects of temperature in atomic magnetic moments. They modeled the magnetic moment arrangement in finite temperatures and provided Curie temperature calculations; the latter are not provided by DMFT. They were able to imitate the atomic moments and critical temperature tendency across the RCo_5_-type isostructural compounds for all lanthanide series plus Y. The tuning of computational parameters in order to match the results with experimental evidence is also a common practice in the literature and provides meaningful insight into the physics of these complex intermetallic systems. Unfortunately, but possibly in correlation with experimental reality, SmCo_5_ seems to be the strongest magnet, especially at elevated temperatures, at least for CaCu_5_-type compounds with a single rare earth.

More complicated stoichiometries in the vicinity of the high-entropy alloy region, Sm_1−x_MM_x_Co_5−y−z_Fe_y_Ni_z_ (*x* = 0–0.7; *y* = 0.5–1.5; *z* = 0.5–1) has also been synthesized and studied both experimentally and theoretically by computational methods [75,88]. The introduction of Fe and Ni simultaneously with Mm did not seem to fully stabilize the hexagonal CaCu_5_-type structure for very high Ce_3_La misch metal contents. Practically single-phase samples have been acquired for 50% replacement of Sm by misch metal and 20% substitution of Co by Fe and Ni in equal concentrations. Magnetic properties are weakened, but slower than the reduction in cost. The Sm_0.5_Mm_0.5_Co_4_Fe_0.5_Ni_0.5_ compound presents a mass magnetization of 85 Am^2^/kg, which is reasonably close to the value of the mother compound. Ab initio calculations confirm experimental results, and the usual higher estimation of magnetization is still present. A positive effect of Ni in the overall thermodynamic stability of the material was observed theoretically, with the trade-off of negative effects in magnetic properties.

## 7. Summary

This review presents the progress on modification of SmCo_5_-type alloys suitable for permanent magnets. SmCo_5_ is the basic material for an important class of permanent magnets and presents enormous uniaxial magnetocrystalline anisotropy and large temperature endurance.

A way to produce new low-cost SmCo5-type compounds is chemical modification, reducing the Co content by substitution with lower-cost elements, or replacing Sm with abundance greater than their demand. An important instrument to achieve these goals are theoretical calculations in predicting the possible stable alloys and their magnetic properties. Partial substitution of Co with *d*-block elements (Sc, Ti, V, Cr, Mn, Fe, Co, Ni, Cu, and Zn) has been widely studied. CaCu_5_-type structures can be stabilized by replacing Co with a limited amount of Fe or Cu or both, mainly in SmCo_1−x_M_x_ (M = Fe, Cu) ribbons. Depending on the mode of preparation, different magnetic properties of SmCo_1−x_M_x_ are reported. The coercivity and magnetization increases for small Fe or Cu atom substitution, then decreases rapidly. When crystallized on a single CaCu_5_-type phase, SmCo_1−x_Fe_x_ ribbons exhibit a coercivity up to 14 kOe, which was gradually decreased when Fe content increased from x = 0.6 to 1.0. SmCo_1−x_Cu_x_ exhibits a much higher coercivity, reaching a value of 27 kOe in a composition range from x = 0.6 to 0.8. However, the nonmagnetic atom Cu decreases the magnetization and Curie temperature drastically.

Researchers often use expensive methods such as induction melting or arc melting in the range of 1300–1400 °C, then annealing the samples at high temperature. Chemical methods are a possible alternative. Using a reduction diffusion to synthesize the materials could be proven cost-efficient when compared to the established metallurgy methods. Cu doping may reduce magnetization, but on the other hand may induce positive changes in the anisotropy energy and coercivity. The cost of this magnet decreases, but the coercivity reaches a modest value of ~7 kOe

The partial substitution of Co with p elements like Al, B, Ga in RCo_5_ (R = Y, Pr, Nd, Sm, Gd, Tb) alloys influences their magnetic properties and structure. The RCo_4_B alloys crystallize in the hexagonal CeCo_4_B-type structure. Although the saturation magnetization *M*_s_ and T_c_ are decreased upon B substitution for Co, the SmCo_4_B compound still possesses excellent magnetic properties and is promising for permanent magnet applications. Substitution of Co by Ga and Al in the RCo_5_ compound maintains the CaCu_5_-type structure of RCo_5_.

The effect of partial Ni substitution for Co on the structural properties of SmCo_5_ has been investigated by computational methods. Energetically favorable atomistic configurations of SmCo5-xNix were found. However, these configurations do not exhibit the maximum magnetization. Replacement of Co by Ni in the SmCo_5_ system does not favor maximum magnetization, while replacement of Fe by Co the SmCo_5_ system is unstable. This controversy was recently addressed by two theoretical studies, where a new stable and efficient SmCoNiFe_3_ permanent magnet was proposed. The magnetic properties of the predicted SmCoNiFe_3_ (energy product, T_c_, Ms) are better than the corresponding properties of Nd_2_F_14_B magnet types. Therefore, SmCoNiFe_3_ can potentially replace SmCo_5_ or Nd-Fe-B types in many applications. Despite these efforts, the experimental synthesis of this predicted magnet has not yet been reported.

The introduction of Ce and La in the SmCo_5_ system attracted attention as early as the discovery of the initial compound. Lately, a recent series of theoretical as well as experimental studies developed the possibility of simultaneously introducing metallic Ce-La at a Ce:La ratio of 3:1 into the SmCo_5_ system and reducing the Co content. The theoretical framework of the compounds and the relevant ab initio parameters were also intensively investigated. Preliminary calculations showed that 50% replacement of Sm by Ce_3_La misch metal was of specific interest.

The simultaneous replacement of Sm by 50% Mm and 20% substitution of Co by Fe and Ni in equal concentration seems to stabilize the hexagonal CaCu_5_-type structure, practically acquiring a single-phase sample. The partial replacement of an expensive rare-earth element by a less expensive one may establish a material that could be used as the basis for a “gap” magnet and may find potential use in applications with low cost.

There is still room in the research field to modify SmCo_5_ alloys. Modification of RCo_4_B by replacing Co with another transition metal or non-transition metal and/or Mm Ce-La could lead to new alloys with low cost and good magnetic properties. The processing of Sm-Mm-Co-M is also an open field of research to produce low-cost permanent magnets.

## Figures and Tables

**Table 1:** Intrinsic magnetic properties of some magnets at 298 K [35] and 523 K [17]: saturation magnetization Ms, anisotropy constant K_1_, coercivity iHc and energy product (BH)_max_.

Material	At 298 K				At 523 K		T_c_ (K)
	Ms	K1	_i_H_c_	(BH)_max_	iHc	(BH)_max_	
	(kG)	(kOe)	(kOe)	(MGOe)	(kOe)	(MGOe)	
Nd_2_Fe_14_B	16.08	13.7	13.9	64.34	0.65	1.52	588
Sm_2_(Co, Fe)_17_ based	12.19	10.69	25	36.95	10.3	25.4	1078
SmCo_5_	10.81	8.95	29	29.08	15.2	15. 8	1020
Alnico 5	14.07	0.74	0.75	5.3	0.75	5.18	1210

**Table 2:** Magnetic properties of Nd_2_Fe_14_B, SmCo_5_ and predicted SmCoNiFe_3_.

Material	M_s_(MA/m)/kG)	T_c_ (K)	K1 (MJ/m^3^)	BH_max_ (kJ/m^3^)/MGOe)
Nd_2_Fe_14_B	1.28/16.8	588	4.9	512/64.34 *
SmCo_5_	0.86/10.81	1020	17.2	231/29.08 *
SmCoNiFe_3_	1.08/13.57	1103	9.2	361/45.37 **

* [35,36], ** [75].

## Data Availability

No new data were created or analyzed in this study. Data sharing is not applicable to this article.
